# Rectal Metastasis of Prostate Cancer: About a Case

**DOI:** 10.4021/jocmr2010.05.309w

**Published:** 2010-06-15

**Authors:** Aurelien Venara, Emilie Thibaudeau, Souhil Lebdai, Stephanie Mucci, Catherine Ridereau-Zins, Rahmene Azzouzi, Antoine Hamy

**Affiliations:** a Digestive Surgery Department, chu Angers, 4 rue Larrey, 49000 Angers, France; b Urology Department, chu Angers, 4 rue Larrey, 49000 Angers, France; c Radiology Department, chu Angers, 4 rue Larrey, 49000 Angers, France

## Abstract

Prostate adenocarcinomas present a high risk of metastasis. We report a case of an atypical prostate cancer metastasis. A male patient presented a prostatic adenocarcinoma treated by surgery. A biological recurrence was discovered during the follow-up by an increased rate of Prostate Specific Antigen (PSA) and was treated by hormonotherapy. Several months later, there was a re-increase of the PSA rate. The CT scan showed a radiation proctitis aspect. An intermittent hormonotherapy was decided. Six months later, he presented abdominal pain. Examinations were performed and showed a rectal carcinosarcoma with prostate origins. A surgical management was realised. The outcomes were an early recurrence. A symptomatic treatment was decided. There are not any rectal localisations reported in the literature. Only loco-regional invasions of the rectum are described and no histological modification of metastasis compared to the primitive tumor has been reported. So, we report a metastasis of a prostate adenocarcinoma which transformed into a carcinosarcoma.

**Keywords:**

Adenocarcinoma; Carcinosarcoma; Metastasis; Prostate; Rectal neoplasm

## Introduction

Prostate adenocarcinomas are highly metastatic cancers. Elective locations are bones. A recent review of the literature about metastatic locations of prostate cancer did not report any isolated rectal metastasis [1]. We describe a case of a 75 years old man who presented such a metastasis.

## Case Report

**Figure 1.**
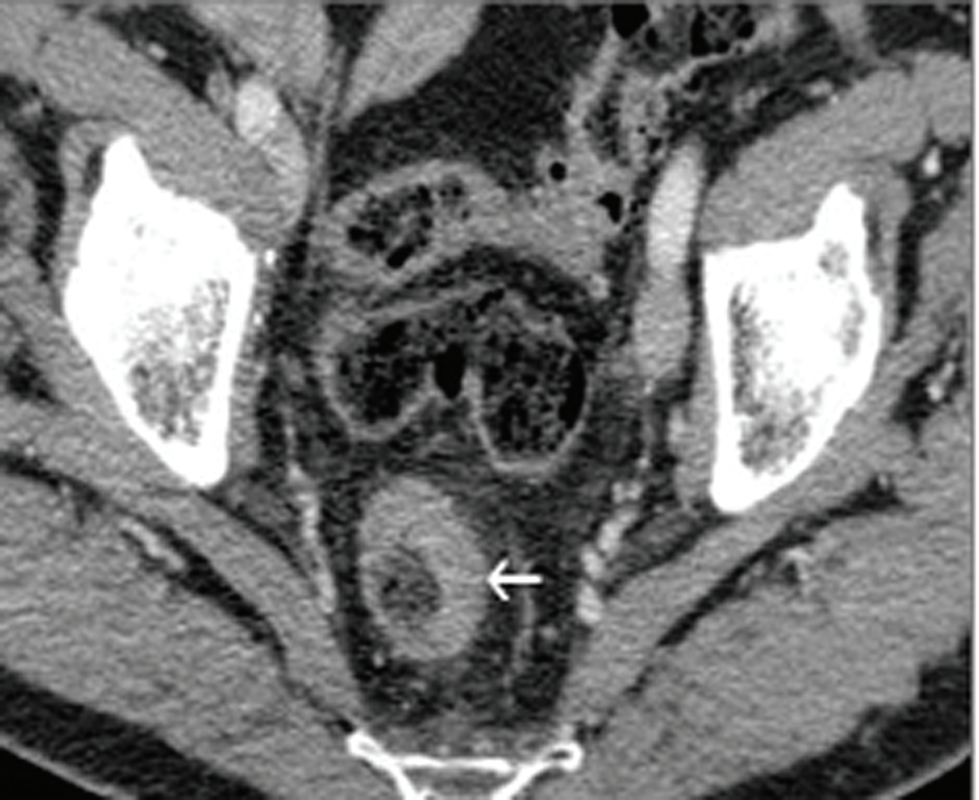
Transversal view of abdominal CT scan. Circumferential thickening of the rectal wall (arrow).

**Figure 2.**
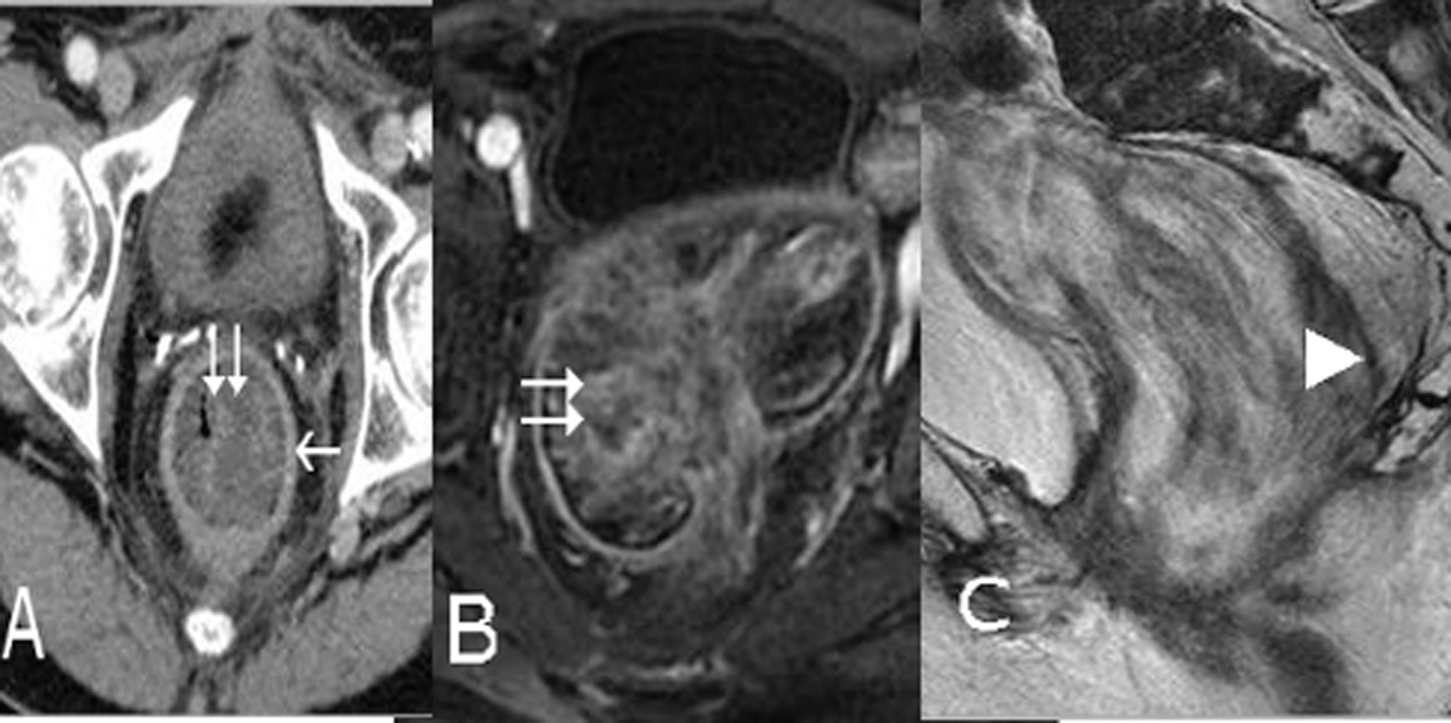
A) Transversal view of abdominal CT scan. Tumor filling of the rectal lumen (double arrow) and circumferential thickening of the rectal wall (arrow). B) RMI, T2-weightened sequence. Heterogeneous, hyper-signal tumor filling the whole rectal lumen (double arrow). C) RMI, T1-weightened sequence, sagittal view. Invasion of the posterior rectal wall (arrow point).

A 75 years old male patient presented a prostate adenocarcinoma in July 1999, confirmed by positive transrectal prostate biopsies and treated by radical prostatectomy, seminal vesicles ablation, bilateral ilio-obturator lymphadenectomy and vesico-uretral junction reconstruction. Histology did not show any node metastasis on the 9 nodes from the ilio-obturator lymphadenectomy. A large adenocarcinoma was found in the prostate with a Gleason score at 3 + 4 with an extra-capsular extension and a multiple perineural extension. The seminal vesicles were healthy. In the end of 2005, during his follow-up, an increased rate of Prostate Specific Antigen (PSA) was discovered. The imaging evaluation was normal. The follow-up of the PSA rate showed a progressive increase reaching 6.85 ng/ml (N < 3). A RMI was performed in April 2006 which did not show any local recurrence. A treatment by hormonotherapy and pelvic radiotherapy were performed which normalized the PSA rate. In March 2008, one year after the end of the treatment, a new re-increase of the PSA at 7.98 ng/ml was found. The imaging did not show any recurrence. Only a circumferential thickening of the rectal wall was found on the pelvic CT scan (Fig. 1). It was interpreted like a radic rectitis. A treatment by intermittent hormonotherapy was then decided. In January 2009, the patient presented abdominal pain with alteration of his general condition. With the occurrence of an occlusive syndrome, an abdominopelvic CT scan was realised. It showed a thickening of the rectal wall with a tumour filling the rectal lumen. The density of the tumour was lower than the walls (Fig. 2A). Rectoscopic biopsies were performed and showed a sarcomatoid carcinoma. An RMI was realised, the T2-weighted sequence showed a thick rectal wall with a heterogeneous rectal tumor in hyper-signal (Fig. 2B). The T1-weighted sequence showed a heterogeneous tumor filling the rectal lumen, fixed to the posterior rectal wall (Fig. 2C). The patient underwent surgery in June 2009. He had an anterior resection of the rectum without continuity reestablishment and thus had a terminal left iliac colostomy instead. The histological analysis found a prostatic carcinosarcoma with intra-parietal focuses of rectal adenocarcinoma. The post-operative period did not present any complication and the pain disappeared. The patient was seen by the surgeon in August 2009. He was healthy and had a PSA rate at 0.23 ng/ml. In September 2009, the patient consulted at the emergency unit for proctorrhagia and rectal syndrome. A proctoscopy was performed on the rectal stump and showed an early recurrence of the carcinosarcoma with the same aspect as previously. A RMI was performed in order to evaluate the tumoral volume. It showed the same type of image as the previous RMI. There was a heterogeneous T2 hyper-signal and T1 hypo-signal tumor filling the whole rectal lumen. The case was discussed in a pluridisciplinary meeting which decided that the management of this patient would be a radiofrequency treatment with comfort-care. This treatment is currently in process.

## Discussion

Prostatic adenocarcinoma presents a high risk of metastasis. The most frequent localization is the bones with more than 90% of the long-term metastasis [1]. Several other localizations were reported including the small intestine (1 - 4%) and the caecum, but never the rectum. A suspicion of metastasis was reported after transrectal prostate biopsy [2], it seemed to be sub-mucosa nodules which have been resected by endoscopy. There was no increase of the PSA rate contrary to our case.

Some autopsy series revealed that 9% of the patients who presented prostate adenocarcinoma had a contiguity invasion of the rectum [3]. In those cases, there were digestive symptoms which could reveal advanced prostate cancer. In our case, we exclude a contiguity invasion because of the delay with the prime surgery, and because of the fact that the adenocarcinoma was encapsulated. However, no rectal invasion was seen during the initial procedure. Thus, it seems to be a rectal metastasis of the prostate adenocarcinoma by distant contamination.

However, the histological differences between the metastasis and the initial tumor imply a mutation of a prostatic adenocarcinoma into a prostatic carcinosarcoma. This mutation could be spontaneous or induced by the radiotherapy.

Carcinosarcoma of the colon is a rare tumour with both epithelial and sarcomatous components. Histogenesis from a common cell progenitor [4] has been reported.

No secondary prostatic carcinosarcomas in the rectum were described in the literature. Also, several cases of prostatic carcinosarcoma were described with metastasis but none was described with metastasis on the colon [5]. Moreover, this case reports an adenocarcinoma transformation into a carcinosarcoma probably induced by the radiotherapy performed for the prostate cancer recurrence treatment.

This case is also the first which reports a metastasis different from the primitive cancer.

Thus, this report presents a double interest: it describes an unusual metastasis site for prostate cancer and also a histological modification of the tumor probably induced by radiotherapy.
